# Two newly recognized species of *Hemidactylus* (Squamata, Gekkonidae) from the Arabian Peninsula and Sinai, Egypt

**DOI:** 10.3897/zookeys.355.6190

**Published:** 2013-11-25

**Authors:** Jiří Šmíd, Jiří Moravec, Lukáš Kratochvíl, Václav Gvoždík, Abdul Karim Nasher, Salem M. Busais, Thomas Wilms, Mohammed Y. Shobrak, Salvador Carranza

**Affiliations:** 1 Department of Zoology, National Museum, Cirkusová 1740, Prague, Czech Republic; 2 Department of Zoology, Faculty of Science, Charles University in Prague, Viničná 7, Prague, Czech Republic; 3 Department of Ecology, Faculty of Science, Charles University in Prague, Viničná 7, Prague, Czech Republic; 4 Faculty of Science, University of Sana’a, Sana’a, Yemen; 5 Biology Department, Faculty of Education, University of Aden, Aden, Yemen; 6 Faculty of Sciences, University of Hail, Hail, Saudi Arabia; 7 Zoologischer Garten Frankfurt, Bernhard-Grzimek-Allee 1, Frankfurt am Main, Germany; 8 Biology department, Faculty of Science, Taif University 888, Taif, Saudi Arabia; 9 Institute of Evolutionary Biology (CSIC-Universitat Pompeu Fabra), Passeig Marítim de la Barceloneta 37–49, Barcelona, Spain

**Keywords:** Reptilia, Gekkonidae, molecular phylogeny, Arabia, Red Sea, *Hemidactylus saba* species group, *Hemidactylus granosus* Heyden, 1827, *Hemidactylus ulii* sp. n.

## Abstract

A recent molecular phylogeny of the Arid clade of the genus *Hemidactylus* revealed that the recently described *H. saba* and two unnamed *Hemidactylus* species from Sinai, Saudi Arabia and Yemen form a well-supported monophyletic group within the Arabian radiation of the genus. The name ‘*Hemidactylus saba* species group’ is suggested for this clade. According to the results of morphological comparisons and the molecular analyses using two mitochondrial (*12S* and *cytb*) and four nuclear (*cmos*, *mc1r*, *rag1*, *rag2*) genes, the name *Hemidactylus granosus* Heyden, 1827 is resurrected from the synonymy of *H. turcicus* for the Sinai and Saudi Arabian species. The third species of this group from Yemen is described formally as a new species *H. ulii* **sp. n.** The phylogenetic relationships of the members of ‘*Hemidactylus saba* species group’ are evaluated and the distribution and ecology of individual species are discussed.

## Introduction

**Figure 1.**
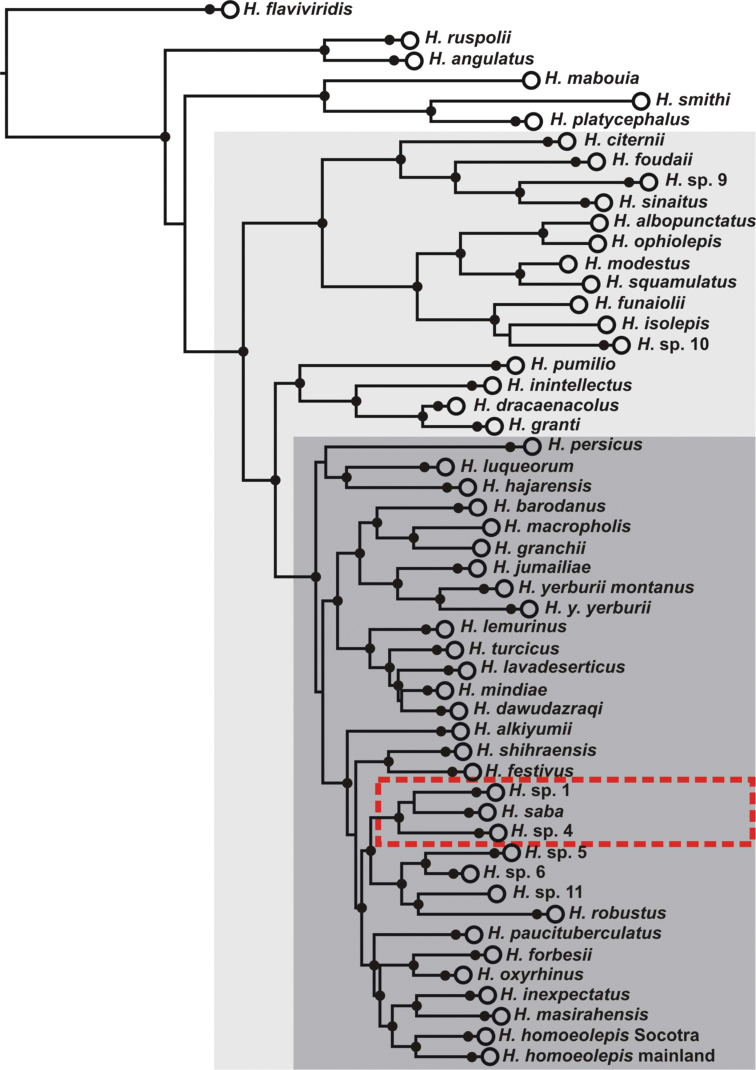
Phylogeny of the *Hemidactylus* Arid clade (light grey rectangle) modified after Šmíd et al. (2013). Dark grey rectangle highlights the Arabian radiation of this clade, dashed red line delimits the ‘*Hemidactylus saba* species group’ dealt with in this study. Black dots indicate ML bootstrap values ≥ 70 and BI posterior probabilities ≥ 0.95.

The genus *Hemidactylus* Oken, 1817, the second most species-rich genus of Gekkonidae (122 currently valid species; Uetz 2013), has been witnessing a species-description boom within the last decade. Eighteen species have been described within the last two years, most of them from the Arabian Peninsula and surroundings areas where 13 new species and a new subspecies have been discovered (Busais and Joger 2011a; Moravec et al. 2011; Torki et al. 2011; Carranza and Arnold 2012). Despite the large number of taxa added recently to the Arid clade of *Hemidactylus* [*sensu* Carranza and Arnold (2006)], it has been shown that the real diversity of *Hemidactylus* in Arabia and northeast Africa is still underestimated, with at least seven species remaining to be described (Busais and Joger 2011b; Moravec et al. 2011; Šmíd et al. 2013). A recent study (Šmíd et al. 2013) revealed that two of these newly recognized but still unnamed species, one from Sinai [labelled in accordance to previous works (Moravec et al. 2011; Šmíd et al. 2013) as *Hemidactylus* sp. 1] and one from Yemen (*Hemidactylus* sp. 4), clustered with the recently described Yemeni endemic *Hemidactylus saba* Busais & Joger, 2011. They form a very well supported clade within the Arabian radiation of the genus (Fig. 1). Although the phylogenetic relationships among these three species were not resolved satisfactorily, it was inferred that they began to diversify approximately 7 million years ago (95% highest posterior density interval 4.3–10), what was followed by a subsequent dispersal of the Sinai species from southern Arabia to the north (Šmíd et al. 2013).

The discovery of a monophyletic species group consisting of one recently described and two newly recognized species calls upon a more thorough study of the nomenclatural status, evolutionary relationships, taxonomy and distribution of its members based on further genetic and morphological data. The present study focuses on this task.

## Material and methods

### Material for phylogenetic analyses

In order to resolve the phylogenetic relationships between the two newly recognized *Hemidactylus* species and *Hemidactylus saba* based on genetic data, a dataset containing only representatives of these three species was assembled. Apart from the data used by Šmíd et al. (2013), additional sequences of the following specimens were produced (Table 1): the holotype and two paratypes of *Hemidactylus saba* (the only known existing material), 21 individuals from Sinai and Saudi Arabia belonging to *Hemidactylus* sp. 1 (Šmíd et al. 2013), and five individuals of the undescribed species from Yemen (*Hemidactylus* sp. 4; Šmíd et al. 2013), one of which was included in the study by Busais and Joger (2011a) (labelled as ‘OTU 7’ therein). Total genomic DNA was extracted using DNeasy Blood & Tissue Kit (Qiagen). Subsequently, sequences for up to two mitochondrial (12SrRNA [*12S*] – ca. 400 bp and cytochrome *b* [*cytb*] – 307 bp) and four nuclear (*cmos* – 402 bp, *mc1r* – 666 bp, *rag1* – 1023 bp, *rag2* – 408 bp) were produced using primers and PCR conditions described in details elsewhere (Šmíd et al. 2013). Chromatograms of all newly obtained sequences were checked by eye and assembled in Geneious 5.6.5 (Biomatters, http://www.geneious.com/​​). All genes were aligned individually using MAFFT (Katoh and Toh 2008) with the iterative refinement algorithm with 1000 iterations. Poorly aligned positions in the alignment of *12S* were eliminated with Gblocks (Castresana 2000) under low stringency options (Talavera and Castresana 2007), producing a final *12S* alignment of 386 bp. Alignments of all coding genes were trimmed so that all started by the first codon position and no stop codons were revealed when translated into amino acids with the appropriate genetic codes.

**Table 1.** List of material used for the phylogenetic analyses. Holotype of *Hemidactylus ulii* sp. n. and *Hemidactylus saba* are in bold. The column ‘Loc. N^o^’ refers to the locality number as shown in Fig. 6.

Species	Code	Museum number	Country	Locality	Loc. N^o^	Lat, Long	*12S*	*cytb*	c*mos*	*mc1r*	*rag1*	*rag2*
*Hemidactylus granosus*	Sher10660	SMB 10660	Egypt	Ayoun Musa	1	29.875, 32.649	JQ957071	JQ957216	JQ957148	JQ957282	-	JQ957409
*Hemidactylus granosus*	Hd41	NMP6V70163/2	Egypt	Sharm el Sheik; Sinai	2	27.885, 34.317	KC818724	HQ833759	JQ957148	-	KC818981	KF647606
*Hemidactylus granosus*	Hd96	NMP6V70163/1	Egypt	Sharm el Sheik; Sinai	2	27.885, 34.317	KC818724	HQ833759	-	-	-	KF647607
*Hemidactylus granosus*	Hd97	NMP6V70163/3	Egypt	Sharm el Sheik; Sinai	2	27.885, 34.317	KC818724	HQ833759	-	-	-	KF647608
*Hemidactylus granosus*	HSA63	ZFMK 94084	Saudi Arabia	Al Wajh	3	26.208, 36.4976	KC818724	HQ833759	KF647576	KF647589	KF647596	KF647610
*Hemidactylus granosus*	HSA64	ZFMK 94085	Saudi Arabia	Al Wajh	3	26.208, 36.4976	KF647571	-	-	-	-	-
*Hemidactylus granosus*	HSA65	ZFMK 94086	Saudi Arabia	15 km S of Al Wajh	4	26.123, 36.5689	KF647570	KF647581	KF647574	KF647590	KF647601	KF647610
*Hemidactylus granosus*	HSA66	ZFMK 94087	Saudi Arabia	15 km S of Al Wajh	4	26.123, 36.5689	KC818724	-	-	-	-	-
*Hemidactylus granosus*	HSA67	ZFMK 94088	Saudi Arabia	15 km S of Al Wajh	4	26.123, 36.5689	KF647569	-	-	-	-	-
*Hemidactylus granosus*	HSA68	TUZC-R8	Saudi Arabia	15 km S of Al Wajh	4	26.123, 36.5689	KF647570	-	-	-	-	-
*Hemidactylus granosus*	HSA69	ZFMK 94089	Saudi Arabia	15 km S of Al Wajh	4	26.123, 36.5689	KF647570	-	-	-	-	-
*Hemidactylus granosus*	HSA70	TUZC-R9	Saudi Arabia	72 km N of Umluj	5	25.614, 36.9867	KF647569	KF647582	JQ957148	KF647591	KF647600	KF647609
*Hemidactylus granosus*	HSA62	TUZC-R10	Saudi Arabia	180 km W of Hail	6	26.883, 40.0874	KF647569	KF647585	JQ957148	KF647588	KF647602	KF647609
*Hemidactylus granosus*	HSA61	IBES10001	Saudi Arabia	Al Ghat	7	26.054, 45.0003	KF647569	KF647585	JQ957148	KF647588	KF647599	KF647610
*Hemidactylus granosus*	HSA57	IBES10183	Saudi Arabia	30 km NE of Alhawiyah	8	21.624, 40.7094	KF647568	KF647580	-	-	KF647597	KF647610
*Hemidactylus granosus*	HSA58	ZFMK 94090	Saudi Arabia	30 km NE of Alhawiyah	8	21.624, 40.7094	KF647569	-	-	-	-	-
*Hemidactylus granosus*	HSA59	TUZC-R11	Saudi Arabia	30 km NE of Alhawiyah	8	21.624, 40.7094	KF647569	-	-	-	-	-
*Hemidactylus granosus*	HSA60	IBES10344	Saudi Arabia	30 km NE of Alhawiyah	8	21.624, 40.7094	KF647569	KF647583	-	-	KF647598	KF647610
*Hemidactylus granosus*	HSA54	IBES10150	Saudi Arabia	20 km S of Ashayrah	9	21.602, 40.6911	KF647568	KF647584	KF647576	KF647588	KF647595	KF647609
*Hemidactylus granosus*	HSA55	ZFMK 94091	Saudi Arabia	20 km S of Ashayrah	9	21.602, 40.6911	KF647569	KF647584	KF647575	KF647588	KF647596	KF647610
*Hemidactylus granosus*	HSA56	IBES10363	Saudi Arabia	20 km S of Ashayrah	9	21.602, 40.6911	KF647569	-	-	-	-	-
*Hemidactylus granosus*	ZFMK 87236	ZFMK 87236	Saudi Arabia	Taif National Wildlife Research Center	10	21.25, 40.96	KF647569	-	-	-	-	-
*Hemidactylus saba*	BJ27	NHM-BS N41914	Yemen	Marib	17	14.9, 45.5	KF647567	-	KF647573	-	-	KF647605
*Hemidactylus saba*	BJ28	NHM-BS N41913	Yemen	Marib	17	14.9, 45.5	KF647567	KF647579	KF647573	KF647586	-	KF647605
***Hemidactylus saba***	**BJ29**	**NHM-BS N41912**	**Yemen**	**Marib**	17	14.9, 45.5	KF647567	-	KF647573	KF647587	KF647594	KF647605
*Hemidactylus ulii* sp. n.	JS48	NMP6V 74834/1	Yemen	Wadi Zabid	11	14.147, 43.517	KC818730	KC818881	KC818789	KC818943	KC819001	KC819062
*Hemidactylus ulii* sp. n.	JS49	NMP6V 74834/2	Yemen	Wadi Zabid	11	14.147, 43.517	KC818731	KC818882	KC818789	-	KF647603	KF647614
*Hemidactylus ulii* sp. n.	JS45	not collected	Yemen	Al Hababi	12	13.333, 43.722	KC818728	KC818878	-	-	-	KF647612
*Hemidactylus ulii* sp. n.	JS46	NMP6V 74833/1	Yemen	Al Hababi	12	13.333, 43.722	KC818728	KC818879	KC818789	-	-	KF647613
***Hemidactylus ulii* sp. n.**	**JS47**	**NMP6V 74833/2**	**Yemen**	**Al Hababi**	12	13.333, 43.722	KC818729	KC818880	KC818789	KC818942	KC819001	KC819061
*Hemidactylus ulii* sp. n.	JS37	NMP6V 74832/1	Yemen	3 km S of Najd an Nashamah	13	13.358, 43.957	KC818727	KC818876	KF647578	KC818943	-	KF647611
*Hemidactylus ulii* sp. n.	JS38	NMP6V 74832/2	Yemen	3 km S of Najd an Nashamah	13	13.358, 43.957	KC818727	KC818877	KC818789	KF647593	-	KF647614
*Hemidactylus ulii* sp. n.	JS32	NMP6V 74835	Yemen	35 km W of Lahij	14	13.032, 44.558	KC818726	KC818875	KC818788	KC818941	KC819000	KC819060
*Hemidactylus ulii* sp. n.	BJ09	NHM-BS N41916	Yemen	Radman	15	14.1, 45.283	KF647572	-	KF647577	KF647592	-	KC819059
*Hemidactylus ulii* sp. n.	JS17	NMP6V 74831/1	Yemen	Al Hadr	16	13.877, 45.8	KC818725	KC818874	KC818787	KC818940	KC818999	KC819059
*Hemidactylus ulii* sp. n.	JS18	NMP6V 74831/2	Yemen	Al Hadr	16	13.877, 45.8	KC818725	-	KC818789	-	KF647604	KC819059
*Hemidactylus angulatus*	JS123	NMP6V 74845/2	Ethiopia	Arba Minch	-	6.034, 37.564	KC818659	KC818807	KC818747	KC818903	KC818956	KC819018
*Hemidactylus flaviviridis*	JS111	not collected	Pakistan	Okara	-	30.811, 73.457	KC818676	KC818822	JQ957126	JQ957253	KC818965	KC819026
*Hemidactylus flaviviridis*	JS113	not collected	India	Haridwar	-	29.964, 78.201	KC818676	KC818823	JQ957126	JQ957253	KC818966	KC819027
*Hemidactylus flaviviridis*	JS119	not collected	Oman	Jalan Bani Bu Hassan	-	22.089, 59.278	JQ957119	JQ957183	KC818754	KC818911	KC818967	KC819028

### Phylogenetic analyses and haplotype networks construction

The final dataset consisted of 36 ingroup individuals. Specimen numbers, localities, and GenBank accession numbers of all genes sequenced are presented in Table 1. The alignment of all concatenated genes was 4012 bp long. The software jModelTest 2.1.1 (Guindon and Gascuel 2003; Darriba et al. 2012) was used to assess the best-fitting model of nucleotide substitution for each gene separately under the Akaike information criterion [AIC, Akaike (1973)]. The best-fitting models were selected as follows: *12S* – GTR+G; *cytb* – GTR+I+G; *cmos* – HKY+I; *mc1r* – TIM2+I; *rag1* – HKY+I; *rag2* – TrN+I). Phylogenetic analyses were performed using maximum likelihood (ML) and Bayesian inference (BI) methods. In order to detect the potential effect of the nuclear genes on the tree topology and nodal support, independent analyses were run on two datasets: (1) a dataset containing mtDNA genes only (*12S*, *cytb*), and (2) a concatenated dataset of all mtDNA and nDNA genes. Sequences of nuclear genes were not phased; heterozygous positions were coded according to the IUPAC ambiguity codes. Gaps were treated as missing data. Three specimens of *Hemidactylus flaviviridis* and one of *Hemidactylus angulatus*, representatives of two different clades of *Hemidactylus* (Carranza and Arnold 2006), were used to root the trees. Uncorrected genetic distances (*p* distances) were calculated in MEGA 5 (Tamura et al. 2011). Almost complete *cytb* sequences (1127 bp) of the new species from Yemen deposited in GenBank (Šmíd et al. 2013) were used to calculate *p* distances within this species, whereas an alignment of 307 bp was used to obtain intraspecific *p* distances within *Hemidactylus saba* and the new species from Saudi Arabia and Sinai, and also interspecific *p* distances between these three species.

Maximum likelihood analyses of both datasets were performed in RAxML 7.0.3 (Stamatakis 2006) using raxmlGUI (Silvestro and Michalak 2012) graphical extension with parameters estimated independently for each partition, GTR+I+G model of nucleotide evolution and a heuristic search with 100 random addition replicates. Support of the tree nodes was assessed by bootstrap analysis with 1000 pseudoreplications (Felsenstein 1985).

The BI analyses were run in MrBayes 3.2.1 (Ronquist et al. 2012). Appropriate equivalents of the best-fitting models were specified to each partition (gene) and all parameters were unlinked across partitions. Analyses were performed with two runs and four chains for each run for 10^7^ generations, with sampling interval of 1000 generations. Appropriate sampling was confirmed by examining the stationarity of log likelihood (ln*L*) values and the value of average standard deviations of the split frequencies. Convergence between two simultaneous runs was confirmed by the PSRF (potential scale reduction factor) value. From 10^4^ sampled trees, 25% were discarded as a burn-in and a majority-rule consensus tree was produced from the remaining ones, with posterior probabilities (pp) of each clade embedded. Nodes with ML bootstrap values ≥ 70% and pp values ≥ 0.95 were considered highly supported (Huelsenbeck and Rannala 2004).

Heterozygous positions in nuclear genes were identified based on the presence of double peaks in chromatograms and using the Heterozygote Plugin in Geneious. For the purpose of haplotype network construction, haplotypes from sequences with more than one heterozygous position were resolved in PHASE 2.1.1 (Stephens et al. 2001). Input data for PHASE were prepared in SeqPHASE (Flot 2010). In order to include as much data as possible, sequences of all *Hemidactylus* species from the Arid clade used in our previous study (Šmíd et al. 2013) were combined with the newly produced sequences and phased together (data not shown). In the case of *rag1*, the original alignment was trimmed to 846 bp, the length at which sequences of all individuals did not contain any N ends that would give misleading results in the allele reconstruction (Joly et al. 2007). PHASE was run under default settings except the probability threshold, which was set to 0.7. Haplotype networks of the four nuclear markers (*cmos*, *mc1r*, *rag1*, *rag2*) were drawn using TCS 1.21 (Clement et al. 2000) with 95% connection limit.

### Material for morphological analyses

Material for morphological comparison included 225 specimens of 8 *Hemidactylus* species and one subspecies (Appendix) and was obtained from the following collections: National Museum Prague, Czech Republic (NMP); Natural History Museum in Braunschweig, Germany (NHM-BS); Senckenberg Forschungsinstitut und Naturmuseum, Frankfurt, Germany (SMF); Zoologisches Forschungsmuseum Alexander Koenig, Bonn, Germany (ZFMK); Museo Civico di Storia Naturale “Giacomo Doria”, Genova, Italy (MSNG); Museo Civico di Storia Naturale di Milano, Milano, Italy (MSNM); Museo Civico di Storia Naturale, Carmagnola, Italy (MCCI); Università di Firenze, Museo Zoologico “La Specola”, Firenze, Italy (MZUF); British Museum of Natural History, London, UK (BMNH); California Academy of Sciences, San Francisco, USA (CAS); Taif University Zoological Collection, Taif, Saudi Arabia (TUZC); Institute of Evolutionary Biology Collection, Barcelona, Spain (IBES); Tomas Mazuch private collection, Dříteč, Czech Republic (TMHC); L. Kratochvíl collection (JEM); J. Šmíd collection (JS); Sherif Baha El Din private collection, Cairo, Egypt (SMB). Names of localities and governorates are spelled according to Google Earth (http://www.google.com/earth/). All coordinates are in WGS84 geographic coordinate system. Table of localities in a CSV text format and high-resolution photographs of all individuals analyzed in this study (397 pictures in total) have been deposited in MorphoBank (Project 1006; http://www.morphobank.org).

### Morphological characters

The following measurements were taken with Powerfix digital calliper to the nearest 0.1 mm: snout-vent length (SVL), measured from tip of snout to vent; head length (HL), measured from tip of snout to retroarticular process of jaw; head width (HW), taken at the widest part of the head; head depth (HD), maximum depth of head; left eye diameter (E), measured horizontally; axilla-groin distance (AG), measured from posterior end of front limb insertion to anterior end of hind limb insertion; tail length (TL), measured from vent to tip of original tail. In addition to these metric characters, the following meristic characters were examined using a dissecting microscope: number of upper and lower labials (left/right); contact of nasals; number of infralabials in contact with first postmentals; mutual position of first postmentals; number of longitudinal rows of enlarged dorsal tubercles; number of lamellae under the first and fourth toe including unpaired proximal ones; and number of preanal pores in males. Terminology and diagnostic characters follow Moravec and Böhme (1997) and Moravec et al. (2011).

## Results

**Figure 2.**
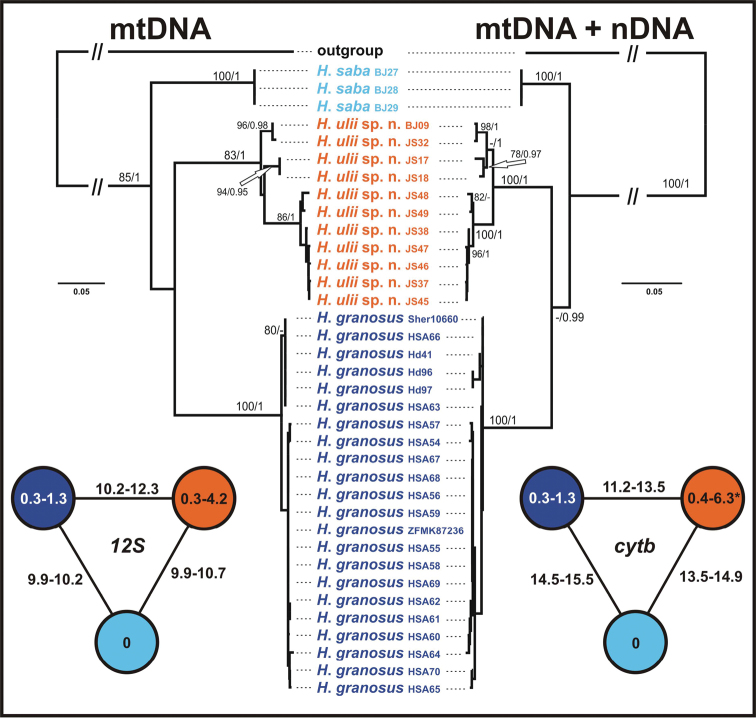
Maximum likelihood trees of mtDNA and mtDNA + nDNA datasets of the ‘*Hemidactylus saba* species group’. ML bootstrap values/Bayesian posterior probabilities are indicated by the nodes. *Hemidactylus flaviviridis* and *Hemidactylus angulatus* were used as outgroups. At the sides, schematic networks showing intra- and interspecific uncorrected *p* distances (in %) in the sequences of *12S* and *cytb*. * intraspecific distances within *Hemidactylus ulii* sp. n. are based on an alignment of 1127 bp, all other values for *cytb* are calculated for an alignment of 307 bp.

**Figure 3.**
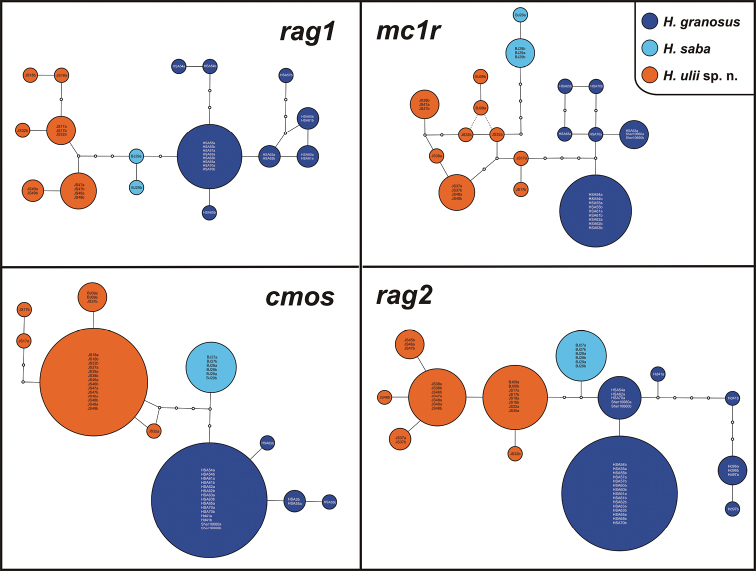
Nuclear allele networks of the four loci analyzed (*cmos*, *mc1r*, *rag1*, *rag2*). Circle sizes are proportional to the number of alleles. Small white circles represent mutational steps. Position of alleles BJ09a and BJ09b in the *mc1r* network is indicated by dashed lines because the sequence of the sample BJ09 (voucher NHM-BS N41916) was 108 bp shorter than the rest of the alignment and haplotype network reconstructions based on both 666 bp and 558 bp alignments linked these alleles to JS32b and JS32a, respectively.

Phylogenetic analyses of both datasets resulted in trees presented in Fig. 2. Tree topology remains congruent with that showed in Šmíd et al. (2013). The three species form a well-supported monophyletic group (mtDNA: ML bootstrap 85/ Bayesian pp 1; mtDNA + nDNA: 100/1) to which we will refer to as the ‘*Hemidactylus saba* species group’ [support of individual species: *Hemidactylus saba* (100/1; 100/1), *Hemidactylus* sp. 1 from Sinai and Saudi Arabia (100/1; 100/1), *Hemidactylus* sp. 4 from Yemen (83/1; 100/1)]. The performed analyses did not resolve the topology within this species group despite the inclusion of more individuals and additional genetic data in comparison with previous works (Moravec et al. 2011; Šmíd et al. 2013). Therefore, with the current knowledge, this group remains polytomic. There is no genetic variability within *Hemidactylus saba* (all three specimens analyzed originate from the same locality) in both of the studied mtDNA genes and a very little variability in nDNA (*mc1r* and *rag1* only) (Fig. 3). The species from Sinai and Saudi Arabia also shows very little variation in mtDNA (intraspecific *p* distance max. 1.3% in both *12S* and *cytb*), but it varies in sequences of all the nDNA genes studied (Fig. 3). On the other hand, the unnamed *Hemidactylus* from Yemen exhibits relatively deep intraspecific differentiation into three well supported lineages. Uncorrected genetic distances between these lineages are up to 6.3% in *cytb* and up to 4.2% in *12S* (Fig. 2). Moreover, the nDNA genes show a high level of genetic differentiation (Fig. 3). Intra- and interspecific genetic distances in both mtDNA genes analyzed between all three species are shown in Fig. 2. The results of the nuclear networks indicate that all alleles for all four independent loci are specific for each species.

The results of the molecular analyses, together with a unique combination of morphological features (see below) confirm the earlier conclusion that the newly recognized *Hemidactylus* sp. 1 and *Hemidactylus* sp. 4 represent two separate species, whose taxonomy and nomenclature need to be resolved.

## Systematics

### Redescription of Hemidactylus granosus

Heyden, 1827

http://species-id.net/wiki/Hemidactylus_granosus

Figs 4 , 5

Hemidactylus granosus Heyden, 1827: p. 17; tab. 5, fig. 1. Lectotype SMF 8723 designated by Mertens (1967); collected by E. Rüppell 1827.
Hemidactylus turcicus (Linnaeus, 1758) – Boettger (1893: 29; part.); Anderson (1898: 80; part.); Salvador (1981: 84; part.); Baha El Din (2006: 66; part.).
Hemidactylus turcicus turcicus (Linnaeus, 1758) – Loveridge (1947: 143; part.); Mertens and Wermuth (1960: 79; part.); Baha El Din (2005: 19; part.); Mertens (1967: 55).
Hemidactylus verrucosus (Cuvier, 1829 [*corr*. *Hemidactylus verrucosus* Gray, 1831]) – Rüppell (1845: 300; part.).
Hemidactylus sp. 1 – Moravec et al. (2011: 24); Carranza and Arnold (2012: 17); Šmíd et al. (2013: 3).
Hemidactylus granosus Terra typica (Heyden 1827): “Egypten, Arabien, und Abyssinien”.
Hemidactylus granosus Terra typica restricta [by lectotype designation by Mertens (1967)]: “Arabia petraea” = Sinai, Egypt.

#### Material examined.

SMF 8723 (lectotype, adult male), Petr. Arabica [Arabia petraea], collected by E. Rüppell in 1827 (MorphoBank M305565–M305594); NMP6V 70163/1 (adult female, MorphoBank M305520–M305528), NMP6V 70163/2 (adult male, MorphoBank M305529–M305542), NMP6V 70163/3–4 (adult females, MorphoBank M305543–M305554, M305555–M305564), Egypt, South Sinai governorate, Sharm el-Sheikh (27.885°N, 34.317°E), ca. 30 m a.s.l., collected by R. Kovář and R. Víta in 1996; ZFMK 94084, ZFMK 94085 (adult females, MorphoBank M305744–M305760, M305761–M305775), Saudi Arabia, Tabuk province, Al Wajh (26.2076°N, 36.4976°E), 5 m a.s.l., 31. V. 2012; ZFMK 94086 (adult female, MorphoBank M305778–M305791), ZFMK 94088, ZFMK 94089 (adult males, M305793–M305799, M305807, M305822–M305827, M305828–M305841), Saudi Arabia, Tabuk province, 15 km S of Al Wajh (26.1226°N, 36.5689°E), 25 m a.s.l., 31. V. 2012; TUZC-R10 (adult female, MorphoBank M305728–M305743), Saudi Arabia, Hail province, 180 km N of Hail (26.8831°N, 40.0874°E), 1020 m a.s.l., 30. V. 2012; IBES10183, TUZC-R11 (adult males, MorphoBank M305656–M305671, M305688–M305701), ZFMK 94090,IBES10344 (adult females, MorphoBank M305672–M305687, M305702–M305717), Saudi Arabia, Makkah province, 30 km NE of Alhawiyah (21.6244°N, 40.7094°E), 1295 m a.s.l., 28. V. 2012; IBES10150, IBES10363 (adult males, MorphoBank M305615–M305628, M305643–M305655), ZFMK 94091 (adult female, MorphoBank M305629–M305642), Saudi Arabia, Makkah province, 20 km S of Ashayrah (21.6022°N, 40.6911°E), 1316 m a.s.l., 28. V. 2012. All Saudi specimens were collected by M. Shobrak, S. Carranza and T. Wilms.

#### Referred material.

SMB 10660, Egypt, Suez governorate, Ayoun Musa (29.875°N, 32.649°E), ca. 12 m a.s.l., collected by S. Baha El Din, date unknown; TUZC-R9, Saudi Arabia, Tabuk province, 72 km N of Umluj (25.614°N, 36.9867°E), 19 m a.s.l., 31. V. 2012; IBES10001, Saudi Arabia, Riyadh province, Al Ghat (26.0545°N, 45.0003°E), 776 m a.s.l., 29. V. 2012; ZFMK 94087, TUZC-R8, Saudi Arabia, Tabuk province, 15 km S of Al Wajh (26.1226°N, 36.5689°E), 25 m a.s.l., 31. V. 2012; ZFMK 87236, Saudi Arabia, Makkah province, Taif National Wildlife Research Center (21.25°N, 40.96°E), 25. VI. 2007 by T. Wilms. These specimens were used for the molecular analyses only.

#### Status and nomenclature.

Heyden (1827) described *Hemidactylus granosus* as a new species occurring in Egypt, Arabia and Abyssinia (Ethiopia and Eritrea). Although not explicitly mentioned by the author, the description was apparently based on four specimens collected by Rüppell currently deposited in the Senckenberg Naturmuseum Frankfurt (collection numbers SMF 8723–8726). Heyden did not diagnose the new species against *Hemidactylus turcicus* (Linnaeus, 1758) and in respect to our today’s knowledge on the morphological variation in *Hemidactylus* the description of *Hemidactylus granosus* is very general. Traditionally, *Hemidactylus turcicus* has been considered a common species widely distributed across the Mediterranean and the Middle East. As the general diagnostic characters of *Hemidactylus granosus* given by Heyden (1827) were also applicable to *Hemidactylus turcicus* at that time, the name *Hemidactylus granosus* Heyden, 1827 was considered its junior synonym (e.g. Boulenger 1885, Loveridge 1947, Mertens and Wermuth 1960, Mertens 1967, Salvador 1981, Baha El Din 2006).

Recent examination (by JŠ) of four specimens collected by Rüppell (SMF 8723–8726) has shown that one of them [SMF 8723 designated by Mertens (1967) as lectotype of *Hemidactylus granosus*; for description see below] corresponds morphologically to *Hemidactylus* sp. 1 from Sinai. The other three specimens from this series morphologically correspond to *Hemidactylus robustus* Heyden, 1827 (SMF 8725, 8726) and *Hemidactylus* cf. *granosus* (SMF 8724), an animal superficially resembling *Hemidactylus granosus* but differing from the members of the ‘*Hemidactylus saba* species group’ in several important characters (see below). These findings lead to the conclusion that *Hemidactylus granosus* Heyden, 1827 is a valid taxon and needs to be resurrected from the synonymy of *Hemidactylus turcicus*. In the light of current knowledge, the range of *Hemidactylus turcicus* does not include a large part of Egypt, being restricted mostly to northern Egypt including Sinai and its Red Sea coast. The species is also missing in Arabia (sensu lato) and Ethiopia (Carranza and Arnold 2006; Moravec et al. 2011; Rato et al. 2011; Šmíd et al. 2013).

#### Diagnosis.

*Hemidactylus granosus* is a member of the ‘*Hemidactylus saba* species group’ within the Arabian radiation of the Arid clade as evidenced by the mtDNA and nDNA analyses. The species has the following combination of molecular and morphological characters: (1) Uncorrected genetic distance from *Hemidactylus saba*: 9.9–10.2% in *12S*, 14.5–15.5% in *cytb*; from *Hemidactylus* sp. 4: 10.2–12.3% in *12S*, 11.2–13.5% in *cytb*; (2) small size, SVL 39.0–53.2 mm in males, 40.6–53.3 mm in females; (3) rather elongated head, head length 24–28% of SVL, head width 68–86% of head length, head depth 33–47% of head length; (4) tail length 107–130% of SVL; (5) uppermost nasals separated by a small shield in 89% of specimens; (6) large anterior postmentals in wide mutual contact, and always in contact with the 1^st^ and 2^nd^ lower labial; (7) 9–11 upper labials; (8) 7–9 lower labials; (9) 14–15 longitudinal rows of enlarged, subtriangular, distinctly keeled dorsal tubercles; (10) 7–8 lamellae under the 1^st^ toe and 10–13 under the 4^th^ toe; (11) ca. 6–8 tail segments bearing 6 pointed tubercles; (12) 4–7 preanal pores in males forming a continuous row on the left and right side; (13) subcaudals enlarged; (14) in life, dorsum pale buff with dark brown spots tending to form transverse bands or X-shaped markings, dark horizontal stripe in prefrontal and temporal region, tail with ca. 10–13 dark brown transverse bands, venter white.

#### Description of the lectotype.

**Figure 4.**
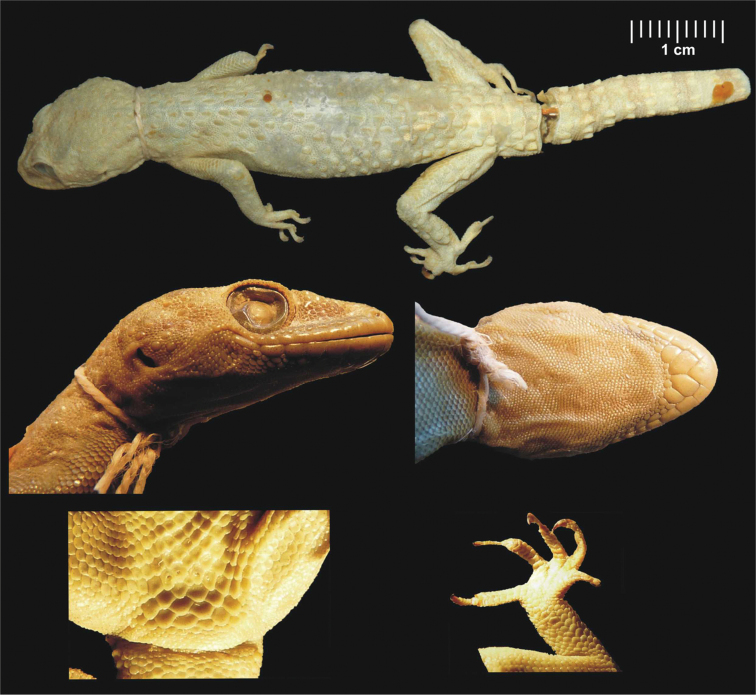
Male lectotype of *Hemidactylus granosus* (SMF 8723) from Sinai, Egypt. General habitus, lateral and ventral view of the head, precloacal region with preanal pores, right hind leg. Scale refers to the uppermost picture only.

**Figure 5.**
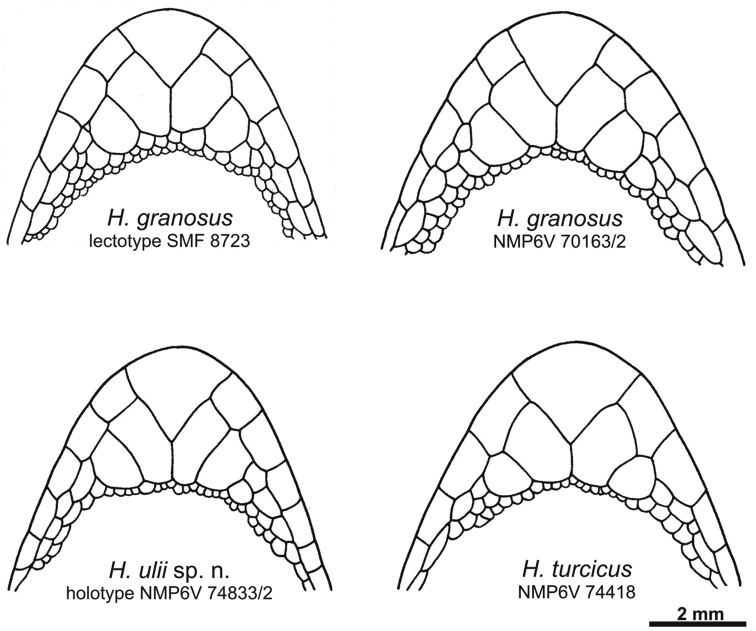
Schematic drawing of the chin region of the lectotype and a new specimen from Sinai of *Hemidactylus granosus*, the holotype of *Hemidactylus ulii* sp. n., and *Hemidactylus turcicus* from Sinai.

SMF 8723, adult male [erroneously determined as female by Mertens (1967)]. Head and body moderately depressed (Fig. 4). Upper labials (10/10), lower labials (8/7). Nostril between rostral, three subequal nasals and in punctual contact with first upper labial. Uppermost nasals separated by a small inserted scale. Mental triangular, as long as wide. Anterior postmentals long, in a broad contact with each other, both in contact with the 1^st^ and 2^nd^ lower labial reaching in about one fourth of the width of the 2^nd^ labial. Second postmentals almost round, touching only the 2^nd^ lower labial (Fig. 5). Two enlarged scales behind each second postmental, the lateral ones in contact with the 3^rd^ lower labial. Eye moderate (E/HL=0.26). Head long, distinctly separated from body by a slender neck. Crescent-shaped ear opening. Interorbital region, crown of head and temporal area above the level of ear opening covered by round smooth tubercles. Dorsal region of the specimen is slightly scarred so it is not possible to count the enlarged tubercles on both sides precisely, but there are seven longitudinal rows of large, keeled and caudally pointed tubercles on the left side from which we infer there were originally 14 rows on both sides together. Lower arms, thighs and lower legs with prominent tubercles without keels. Tail original with 6 segments bearing 6 pointed tubercles, broken into three pieces, subcaudals enlarged from just after the hemipenial bulges. Lamellae under the 1^st^ toe 7/7, lamellae under the 4^th^ toe 11/11. Four preanal pores in a continuous row. No femoral pores or enlarged femoral scales. Colour (in alcohol) faded due to long fixation.

Measurements (in mm): SVL 51.5, HL 12.9, HW 9.8, HD 6.0, E 3.3, AG 23.7.

Paralectotype SMF 8724 differs from other individuals of *Hemidactylus granosus* in having relatively high head (HD 50% of HL), lower number of lower labials (6), uppermost nasals in wide contact, first postmentals in contact with 1^st^ lower labials, and 2 preanal pores.

#### Comparison.

*Hemidactylus granosus* can be distinguished from other member of the ‘*Hemidactylus saba* species group’ and from other congeners distributed in Sinai and the Red Sea coast by the following set of characters (see also Table 2).

**Table 2.** Morphological comparison among members of the ‘*Hemidactylus saba* species group’ and with other *Hemidactylus* species from Sinai and SW Yemen. The values are given as follows: sample size, mean ± standard deviation above, min. – max. value below.

Species / Character	*Hemidactylus saba* species group						*Hemidactylus robustus*		*Hemidactylus turcicus*		*Hemidactylus mindiae*		*Hemidactylus jumailiae*		*Hemidactylus yerburii yerburii*		*Hemidactylus yerburii montanus*	
	*Hemidactylus granosus*		*Hemidactylus saba*		*Hemidactylus ulii* sp. n.													
Upper labials	18	9.4 ± 0.5	3	9.3 ± 0.8	10	9.3 ± 0.8	27	9.4 ± 0.7	33	8.2 ± 0.5	5	10.8 ± 0.8	18	9.8 ± 0.7	51	10.3 ± 0.7	57	10.2 ± 0.7
		9–11		8–10		8–10		8–11		7 - 10		10 - 12		8–12		9–12		8–12
Lower labials	18	7.4 ± 0.4	3	7.7 ± 0.6	10	8.0 ± 0.6	27	7.7 ± 0.6	33	6.7 ± 0.5	5	8.1 ± 0.4	18	8.2 ± 0.6	51	7.9 ± 0.5	57	7.8 ± 0.6
		7–9		7–8		7–9		6–9		6–8		7–9		7–10		6–9		6–10
Nasals in contact (%)	18	11	3	33.3	10	40	27	22.2	33	21.2	5	0	18	5.5	51	7.8	57	5.3
1^st^ postmental in contact with 2^nd^ lower labial (%)	18	100	3	33.3	10	100	27	70.3	33	12.1	5	80	18	83.3	51	98	57	89.5
Rows of dorsal tubercles	18	14.1 ± 0.2	3	14 ± 0.0	10	14.1 ± 1.0	27	14.8 ± 1.2	33	13.8 ± 0.7	5	12.4 ± 0.9	15	14 ± 1.4	46	15.3 ± 1.1	53	15.2 ± 1.2
		14–15		14–14		12–16		13–18		12–16		12–14		12–16		13–18		12–18
Pores	8	5.6 ± 1.1	1	6	2	8 ± 0.0	9	6.1 ± 0.8	13	7.2 ± 1.4	1	4	9	7.2 ± 1.1	23	13.7 ± 2.2	27	11.2 ± 1.1
		4–7				8 - 8		5–8		6–10				6–9		10–18		9–13
Lamellae under 1^st^ toe	18	7.4 ± 0.5	3	8.2 ± 0.3	10	5.4 ± 0.5	27	6.1 ± 0.5	32	6.5 ± 0.5	5	6.2 ± 0.3	18	6.9 ± 0.7	51	6.7 ± 0.4	57	6.3 ± 0.4
		7–8		8–9		5–6		5–8		6–7		6–7		6–8		6–8		5–7
Lamellae under 4^th^ toe	18	11.5 ± 0.7	3	11.2 ± 0.3	10	8.6 ± 0.5	27	10.1 ± 0.7	32	9.7 ± 0.6	5	10 ± 0.0	18	10.9 ± 0.8	51	10.4 ± 0.6	57	10.2 ± 0.5
		10 - 13		11–12		8–9		8–12		8–11		10–10		9–12		9–12		9–11
SVL (males)	8	46.8 ± 5.9	1	58.3	2	38.6 ± 2.6	8	41.8 ± 2.3	13	46.0 ± 5.8	1	49.3	8	48.4 ± 4.1	23	58.5 ± 7.1	25	56.5 ± 5.7
		39.0–53.2				36.8–40.4		37.0–43.7		37.3–54.1				40.0–54.2		43.6–74.9		45.2–65.3
SVL (females)	10	49.0 ± 3.5	2	53.5 ± 7.9	2	40.1 ± 0.9	16	43.6 ± 4.7	18	49.2 ± 5.1	4	46.2 ± 11.4	8	48.6 ± 3.3	23	55.7 ± 5.3	30	52.6 ± 5.1
		40.6–53.3		47.9–59.1		39.4–40.7		32.7–50.1		39.4–56.2		35.6–56.6		43.1–54.0		43.6–62.1		42.4–64.1

From *Hemidactylus saba* by having distinctly keeled dorsal tubercles (smooth in *Hemidactylus saba*), and lower number of lamellae under the 1^st^ toe (7–8 vs. 8–9).

From *Hemidactylus* sp. 4 (described below) by its larger size (max. SVL 53.2 mm vs. 40.4 mm in males, 53.3 mm vs. 40.7 mm in females), in having more frequently separated uppermost nasals (100% vs. 60% of specimens), lower number of preanal pores in males (4–7 vs. 8), and higher number of lamellae under the 1^st^ (7–8 vs. 5–6) and 4^th^ (10–13 vs. 8–9) toe.

From *Hemidactylus flaviviridis* by its smaller size (max. SVL 53.2 mm in males and 53.3 mm in females vs. up to 90 mm [Anderson (1999); sexes not distinguished]), by the presence of enlarged dorsal tubercles, and the absence of femoral pores in males.

From *Hemidactylus mindiae* by the lower number of supralabials (9–11 vs. 10–12), by having anterior postmentals in wide contact (punctual in *Hemidactylus mindiae*) and keeled dorsal tubercles (smooth in *Hemidactylus mindiae*).

From *Hemidactylus robustus* by the larger size of males (max. SVL 53.2 mm vs. 43.7 mm), longer tail (tail length 53.0–64.8 mm vs. 40.9–48.7 mm), and lower number of preanal pores in males (4–7 vs. 5–8).

From *Hemidactylus turcicus* by its higher number of upper labials (9–11 vs. 7–10), in having anterior postmentals more frequently in contact with 2^nd^ lower labial (100% vs. 12.1%), in having anterior postmentals in wide mutual contact behind the mental scale (contact punctual in 67% specimens of *Hemidactylus turcicus*), and by the lower number of preanal pores in males (4–7 vs. 6–10).

#### Variation.

**Figure 6.**
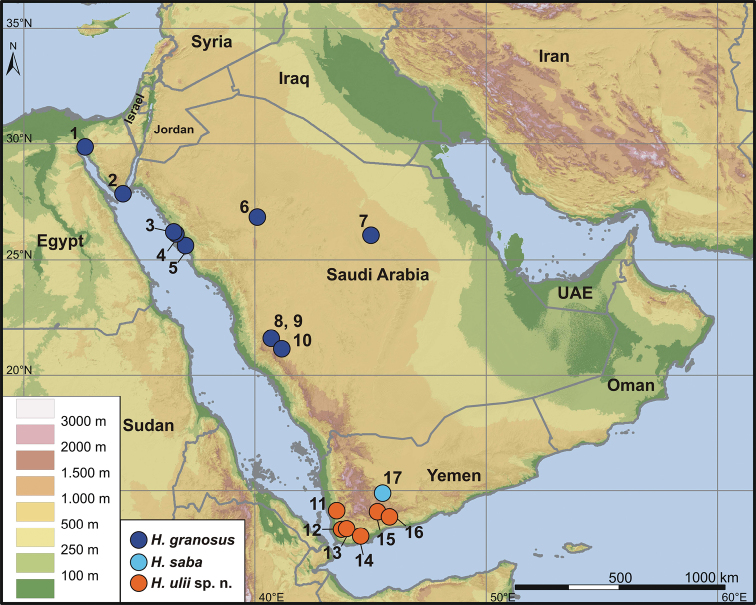
Distribution map of *Hemidactylus granosus*, *Hemidactylus saba* and *Hemidactylus ulii* sp. n. For the list of locality names and their corresponding numbers in the map see Table 1.

Specimens with intact tail vary in number of tail segments bearing 6 pointed tubercles (7–8). The original portion of the tail of the female NMP6V 70163/4 is very wide at the base, separated from cloacal region by a basal constriction. One specimen (IBES10212) is the only animal with 15 longitudinal rows of enlarged tubercles. Another one (IBES10284) has uppermost nasals in wide contact. Most striking is the variation in the number of preanal pores in males. Whereas the lectotype and the only male from Sinai (NMP6V 70163/2) have both 4 pores, all males from Saudi Arabia have 6–7 pores. There seems to be clinal variability in this character, males from NW of the known range (Fig. 6) possess only 4 preanal pores, all animals from the eastern Red Sea coast in Saudi Arabia have 6 pores and a single individual from the southern limit of the range has 7 pores.

**Figure 7.**
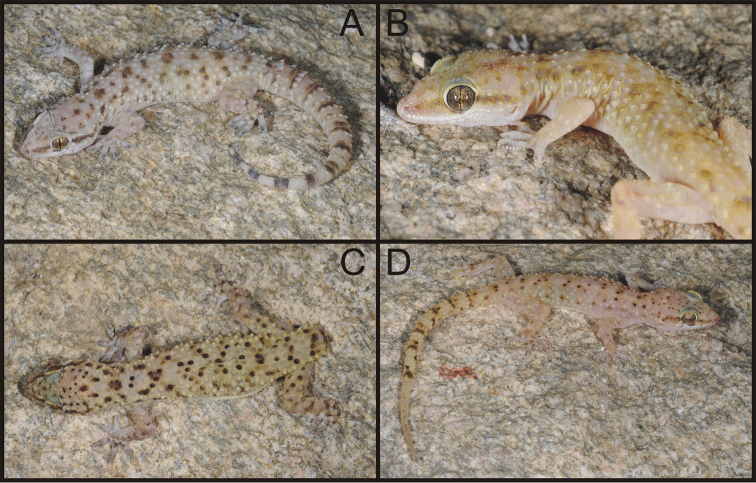
Live specimens of *Hemidactylus granosus* from Saudi Arabia. **A** IBES10344, 30 km NE of Alhawiyah (loc. number 8) **B** TUZC-R10, 180 km W of Hail (6) **C** ZFMK 94091, 20 km S of Ashayrah (9) **D** ZFMK 94086, 15 km S of Al Wajh (4).

Coloration (in life) pale buff dorsally (Fig. 7). Conspicuous dark brown horizontal stripe in loreal and temporal area, terminated at the level of ear from where it continues in a series of dark patches on the neck. Four barely visible X-shaped markings on dorsum formed mainly by dark brown enlarged tubercles (first on nape, second across scapulae, third in lumbal region, and fourth just in front of the anterior insertion of hind limbs). Isolated dark brown stripe runs across body in the place of posterior insertion of hind limbs. Regenerated tails are uniformly buff from above. Dorsum, sides of chin, underside of front and hind limbs and underside of tail with faint stipple visible under magnification. Belly white. Tips of fingers and toes black behind insertion of terminal phalanges. Coloration is consistent among all specimens and varies only in distinctness of the markings.

There is a very low variation in mtDNA between specimens from Sinai and Saudi Arabia (max. 1.3% in both *12S* and *cytb*). All animals from Sinai share the same haplotypes in *12S* and also *cytb* gene. All four nuclear loci studied show some degree of intraspecific variation (Fig. 3).

#### Distribution and ecology.

Eduard Rüppell collected the original series in 1827 when he began his marine biological studies of the Red Sea and travelled from Egypt to Eritrea. There is no specific information that he went to Arabia as well (Rüppell 1826–1828; Klausewitz 2002; Wagner 2008); therefore the original distribution of *Hemidactylus granosus* described as “Egypt, Arabia, and Abyssinia [Ethiopia and Eritrea]” by Heyden (1827) was probably too general and incorrect. Because there were no other specimens assignable with certainty to *Hemidactylus granosus* apart from the four individuals collected in Sinai (SMF 8723–8726, for their current status see ‘Status and nomenclature’ section) (Boettger 1893), one of which became the lectotype after Mertens’ (1967) designation, Sinai could be considered the only reliable locality for *Hemidactylus granosus*. Here, *Hemidactylus granosus* is also confirmed from two coastal localities in south and west Sinai and from coastal and inland regions in western and central Saudi Arabia (Fig. 6). Nevertheless, a wider distribution of the species along the Red Sea coast can be expected. According to Baha El Din (2005), *Hemidactylus* geckos inhabiting the interior lowland of Sinai and the Eastern Desert in Egypt stand out in having notably coarse scalation. Interestingly, the areas with occurrence of animals with coarse scalation correspond with the presence of individuals with low numbers of preanal pores (Baha El Din 2005), which is typical for the Sinai populations of *Hemidactylus granosus*.

In 1996, when the NMP specimens were collected, the locality in Sharm el-Sheikh was formed by a crop field supplied with drain water from nearby habitations. Geckos were found during the day under unused empty barrels and also inside buildings. Other species syntopic with *Hemidactylus granosus* in Sharm el-Sheikh were: *Hemidactylus turcicus*, *Chalcides ocellatus* (Forskål, 1775), *Stenodactylus sthenodactylus* (Lichtenstein, 1823), and *Ptyodactylus hasselquistii* (Donndorff, 1798) (R. Víta in litt, 2013). However, when visited again in 2010, the locality had changed dramatically (R. Víta in litt, 2013). The whole area was under heavy development and the irrigation channels had disappeared. The current conditions at the place are unknown to us. In 2011 JM surveyed a neighbouring urban area east of this locality. It was covered by a mosaic of tourist resorts and abandoned ruderal plots. In dry anthropogenic habitats (e.g. rubbish dumps, road ditches, old walls and buildings, abandoned construction sites, natural but heavily disturbed open areas, etc.) dominated two very abundant gecko species. *Ptyodactylus hasselquistii* occupied primarily various vertical surfaces whereas *Cyrtopodion scabrum* (Heyden, 1827) prevailed on the ground. *Tropiocolotes nattereri* Steindachner, 1901 was found in dry and relatively well-preserved natural places. *Hemidactylus turcicus* was occasionally encountered in more humid artificial habitats in parks and hotel gardens. Specimens from Saudi Arabia were mostly collected during the day inside concrete tunnels under roads. In some of the tunnels they were syntopic with *Ptyodactylus hasselquistii*. One specimen was also collected on the walls of the Taif National Wildlife Research Centre, where it was also syntopic with *Ptyodactylus hasselquistii*.

### Hemidactylus ulii sp. n.

http://zoobank.org/8E15D1BC-5D4D-4A55-AFEB-2E20FAD40112

http://species-id.net/wiki/Hemidactylus_ulii

Figs 5 , 7 , 8

Hemidactylus turcicus – Rösler and Wranik (1998: 120; part.).
Hemidactylus sp. ‘OTU7’ – Busais and Joger (2011a: 27); Busais and Joger (2011b: 268); Carranza and Arnold (2012: 95).
Hemidactylus sp. 4 – Moravec et al. (2011: 25); Šmíd et al. (2013: 3).

#### Holotype.

NMP6V 74833/2,adult male (MorphoBank M305892–M305902), Yemen, Ta’izz governorate, Al Hababi (13.333°N, 43.722°E), 463 m a.s.l.; collected by L. Kratochvíl, 28. X. 2007.

#### Paratypes.

NMP6V 74833/1 (adult male, MorphoBank M305884–M305891), same collecting data as holotype; NMP6V 74831/1–2 (one adult and one subadult female, MorphoBank M305854–M305863, M305864–M305870), Yemen, Abyan governorate, Al Hadr (13.877°N, 45.8°E), 1151 m a.s.l., collected by L. Kratochvíl on 22. X. 2005; NMP6V 74832/1–2 (two subadult females, MorphoBank M305871–M305875, M305876–M305883), Yemen, Ta’izz governorate, ca. 3 km S of Najd an Nashamah by road (13.358°N, 43.957°E), 1182 m a.s.l., collected by L. Kratochvíl on 26. X. 2007; NMP6V 74834/1–2 (one adult and one subadult female, MorphoBank M305903–M305911), Yemen, Dhamar governorate, Wadi Zabid (14.147°N, 43.517°E), 292 m a.s.l., collected by L. Kratochvíl on 29. X. 2007; NHM-BS N41916 (juvenile, MorphoBank M305842–M305852), Yemen, Al Bayda’ governorate, Radman (14.1°N, 45.283°E), collected by W. Mustafa on 13. XI. 2007.

#### Referred material.

NMP6V 74835 (juvenile), Yemen, Lahij governorate, wadi 35 km W of Lahij (13.032°N, 44.558°E), 297 m a.s.l., collected by L. Kratochvíl on 25. X. 2007; JEM476 (juvenile), same collecting data as holotype; All juvenile specimens were used for comparison of meristic characters and included in the molecular analyses.

#### Diagnosis.

A small species of the ‘*Hemidactylus saba* species group’ withinthe Arabian radiation of the Arid clade of *Hemidactylus*, as evidenced by the mtDNA and nDNA analyses. The new species is characterized by the following combination of molecular and morphological characters: (1) Uncorrected genetic distances from *Hemidactylus saba*: 9.9–10.7% in *12S*, 13.5–14.9% in *cytb*; from *Hemidactylus granosus*: 10.2–12.3% in *12S*, 11.2–13.5% in *cytb*; (2) small size with a maximum recorded SVL 40.7 mm (36.8–40.4 mm in males, 39.4–40.7 mm in females); (3) moderately robust head, head length 28–30% of SVL, head width 70–75% of head length, head depth 37–46% of head length; (4) tail length 116% of SVL (only 1 specimen with intact tail); (5) uppermost nasals separated by a small shield (60% specimens) or in wide contact (40%); (6) large anterior postmentals in wide mutual contact in 90% of individuals, and in contact with the 1^st^ and 2^nd^ lower labial (scarcely and unilaterally with the 1^st^ lower labial only); (7) 8–10 upper labials; (8) 7–9 lower labials; (9) dorsum with 12-16 longitudinal rows of enlarged, slightly keeled, conical tubercles; (10) 5–6 lamellae under the 1^st^ toe and 8–9 lamellae under the 4^th^ toe; (11) ca. 6–8 tail segments bearing 6 tubercles; (12) 8 preanal pores in one continuous row in males; (13) subcaudals enlarged; (14) in alcohol dorsum brownish grey with a pattern of more or less conspicuous dark transverse bands starting on the nape, tail with 9 dark brown transverse bands.

#### Comparison.

*Hemidactylus ulii* sp. n. can be distinguished from the other members of the ‘*Hemidactylus saba* species group’ and from all other congeners distributed in the region by the following combination of characters (see also Table 2):

From *Hemidactylus granosus* by its smaller size (max. SVL 40.4 mm vs. 53.2 mm in males, 40.7 mm vs. 53.3 mm in females), by having less frequently separated uppermost nasals (60% vs. 89% of specimens), higher number of preanal pores in males (8 vs. 4–7), and lower number of lamellae under the 1^st^ (5–6 vs. 7–8) and 4^th^ (8–9 vs. 10–13) toe.

From *Hemidactylus saba* by its smaller size (max. SVL 40.4 mm vs. 58.3 mm in males, 40.7 mm vs. 59.1 mm in females), higher number of preanal pores in males (8 vs. 6), and lower number of lamellae under the 1^st^ (5–6 vs. 8–9) and 4^th^ (8–9 vs. 11–12) toe.

From *Hemidactylus flaviviridis* by its smaller size (maximum SVL 40.4 mm in males, 40.7 mm in females vs. up to 90 mm [Anderson (1999); sexes not distinguished]), the presence of enlarged dorsal tubercles, and the absence of femoral pores in males.

From *Hemidactylus jumailiae* by its smaller size (max. SVL 40.4 mm vs. 54.2 mm in males, 40.7 mm vs. 54.0 mm in females), lower frequency of separated uppermost nasals (60% vs. 95%), in having conical and at least slightly keeled dorsal tubercles (vs. non-protruding and smooth tubercles), and lower number of lamellae under the 1^st^ (5–6 vs. 6–8) and 4^th^ (8–9 vs. 9–12) toe.

From *Hemidactylus robustus* by its smaller size (max. SVL 40.4 mm vs. 43.7 mm in males, 40.7 mm vs. 50.1 mm in females), and lower number of lamellae under the 4^th^ toe (8–9 vs. 8–12).

From *Hemidactylus sinaitus* by the presence of enlarged tile-like subcaudals and in having separated uppermost nasals (60% vs. 9% of specimens).

From *Hemidactylus yerburii montanus* by its smaller size (maximum SVL 40.4 mm vs. 65.3 mm in males, 40.7 mm vs. 64.1 mm in females), lower number of preanal pores in males (8 vs. 9–13), and lower number of lamellae under the 4^th^ toe (8–9 vs. 9–11).

From *Hemidactylus yerburii yerburii* by its smaller size (maximum SVL 40.4 mm vs. 74.9 mm in males, 40.7 mm vs. 62.1 mm in females), lower number of supralabials (8–10 vs. 9–12), lower frequency of having separated uppermost nasals (60% vs. 92%), lower number of preanal pores in males (8 vs. 10–18), and lower number of lamellae under the 1^st^ (5–6 vs. 6–8) and 4^th^ (8–9 vs. 9–12) toe.

#### Description of holotype.

**Figure 8.**
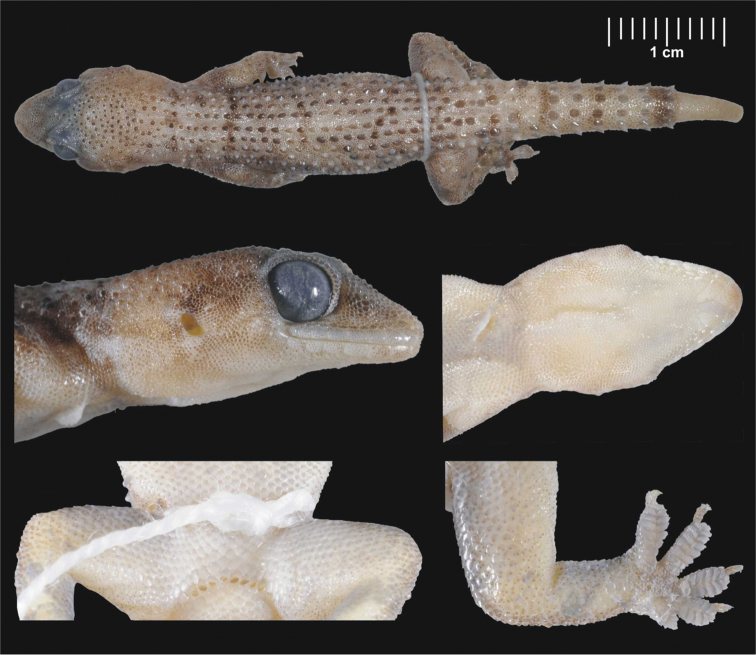
Holotype of *Hemidactylus ulii* sp. n. (NMP6V 74833/2, male) from Al Hababi, Yemen. General habitus, lateral and ventral view of the head, precloacal region with preanal pores, right hind leg. Scale refers to the uppermost picture only.

NMP6V 74833/2, adult male. Body slightly depressed to cylindrical (Fig. 8). Upper labials 8/8, lower labials 7/7. Nostril between rostral, three nasals and in punctual contact with the first upper labial. Uppermost nasals separated by a small inserted shield. Mental almost triangular. Anterior postmentals large and very long, in wide mutual contact behind mental, in contact with the 1^st^ lower labial (left) and the 1^st^ and 2^nd^ lower labials (right) (Fig. 5). Posterior postmentals smaller, in contact with the 1^st^ and 2^nd^ (left) and the 2^nd^ (right) lower labial. Eye moderate (E/HL=0.24). Supraciliar granules with prominent projections, which form a comb-like structure above the eyes. Parietal and temporal region covered with round pointed regularly distributed tubercles. Ear opening oval. Dorsum with 14 longitudinal rows of enlarged, prominent, caudally pointed tubercles bearing distinct longitudinal keels. Thighs and lower legs with scattered enlarged tubercles. Tail partially regenerated from about half of its original length (estimate), original part relatively thick without basal constriction. Conical and keeled tail tubercles on tail segments forming regular whorls. Each whorl separated from the next one by four small scales. Subcaudals enlarged, tile-like. Regenerated part of the tail with small uniform scales without tubercles. Lamellae under the 1^st^ toe 6/6, lamellae under the 4^th^ toe 8/8. Eight preanal pores, no femoral pores or enlarged femoral scales.

Measurements (in mm): SVL 40.4, HL 11.5, HW 8.6, HD 5.2, E 2.8, AG 16.2.

#### Coloration of holotype in preservative.

Overall dorsal coloration brownish grey. An indistinct dark horizontal stripe in loreal and temporal area. Seven dark brown transverse bands across the nape and body, the one in scapular region being the most conspicuous. Dark brown bands also on the original part of the tail. Belly whitish.

#### Variation.

**Figure 9.**
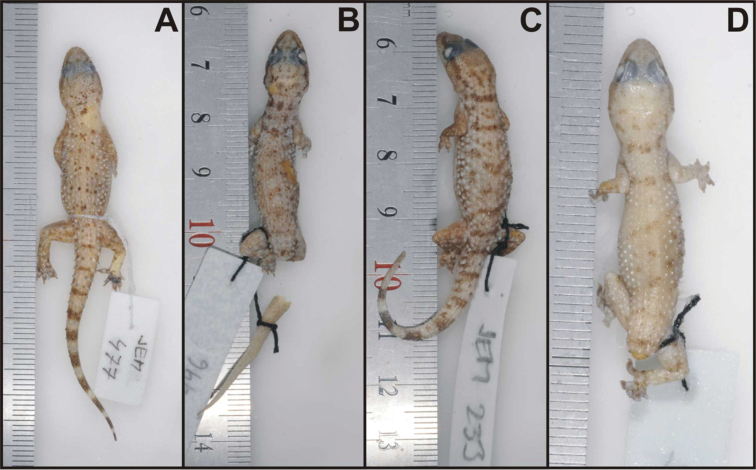
Four (out of eight) paratypes of *Hemidactylus ulii* sp. n. **A** NMP6V 74833/1, male **B** NMP6V 74834/1, female **C** NMP6V 74831/1, female **D** NMP6V 74832/1, subadult female.

The paratypes (Fig. 9) differ from the holotype in the following features: number of upper labials 8–10; number of lower labials 7–9; four paratypes (NMP6V 74831/1, NMP6V 74832/1–2, NMP6V 748333/1) have uppermost nasals in wide contact; anterior postmentals in contact with 2^nd^ lower labials on both sides (except of NMP6V 74832/1 where the arrangement is the same as in the holotype); longitudinal rows of enlarged tubercles 12–16; lamellae under the 1^st^ toe 5–6, lamellae under the 4^th^ toe 8–9. The intact tail of the paratype NMP6V 74833/1 has 7 segments bearing at least six enlarged spine-like tubercles and 9 dark brown transverse bands widening towards the tail tip.

Measurements of paratypes (in mm): NMP6V 74831/1: SVL 40.7, HL 11.5, HW 8.2, HD 4.9, E 3.0, AG 19.0; NMP6V 74831/2: SVL 32.0, HL 9.3, HW 6.6, HD 3.7, E 2.1, AG 12.7; NMP6V 74832/1: SVL 32.7, HL 9.7, HW 7.0, HD 3.4, E 2.3, AG 14.3; NMP6V 74832/2: SVL 32.9, HL 9.3, HW 6.7, HD 3.6, E 2.4, AG 13.5; NMP6V 74833/1: SVL 36.8, HL 10.7, HW 8.0, HD 4.5, E 2.4, AG 14.1, TL 42.5; NMP6V 74834/1: SVL 39.4, HL 11.1, HW 8.1, HD 4.4, E 2.7, AG 16.7; NMP6V 74834/2: SVL 32.0, HL 9.5, HW 6.7, HD 3.9, E 2.5, AG 13.8; NHM-BS N41916: juvenile, not measured.

As already mentioned (Results), the level of genetic variability within *Hemidactylus ulii* sp. n. is very high. The species is divided into three well supported sublineages which reflect the geographic origin of the samples. Although there is a certain geographic separation corresponding with these sublineages, the exact limits are not distinct and also morphological variation among paratypes is not congruent with geography.

#### Etymology.

The species epithet “*ulii*” is a patronym for Prof. Ulrich Joger, a German herpetologist known as Uli among friends, in recognition of his important contribution to the knowledge of the herpetofauna of the Western Palearctic.

#### Distribution and ecology.

*Hemidactylus ulii* sp. n. is known from inland mid-altitude areas (292–1182 m) of southwestern Yemen (Fig. 6). Most specimens were collected in open dry wadis with scattered rocks and boulders, in stony deserts and also in the vicinity of villages in gardens and irrigated cropland fields.

The following reptile specieswere found to occur in sympatry with *Hemidactylus ulii*: *Bunopus spatalurus* Anderson, 1901; *Hemidactylus yerburii yerburii* Anderson, 1895; *Pristurus crucifer* (Valenciennes, 1861); *Pristurus flavipunctatus* Rüppell, 1835; *Pristurus rupestris* Blanford, 1874; *Ptyodactylus* sp.; *Tropiocolotes scorteccii* Cherchi and Spano, 1963; *Acanthodactylus* sp.; *Chamaeleo arabicus* Matschie, 1893; *Pseudotrapelus sinaitus* (Heyden, 1827); *Trapelus flavimaculatus* Rüppell, 1835; and *Pelomedusa subrufa* (Bonnaterre, 1789).

## Discussion

Previous phylogenetic studies of the Arid clade of *Hemidactylus* disclosed an extraordinarily rich diversity within this genus in the Arabian Peninsula (Moravec et al. 2011; Carranza and Arnold 2012; Šmíd et al. 2013). The latter work, besides of showing the phylogenetic relationships among individual species of the Arid clade, highlighted the high level of genetic differentiation and existence of several yet undescribed taxa within this genus. The ‘*Hemidactylus saba* species group’ as defined herein represents one of the monophyletic groups within the Arabian radiation. All three species forming this group – *Hemidactylus granosus*, *Hemidactylus saba*, and *Hemidactylus ulii* sp. n.– are well defined and distinguishable both genetically and morphologically from each other, as well as from other *Hemidactylus* species that occur in the same area. Geographically, *Hemidactylus saba* and *Hemidactylus ulii* sp. n. are confined to the foothills and submontane areas of southwestern Yemen, where they occupy mid-altitude elevations (292–1182 m in *Hemidactylus ulii* sp. n., 1180 m in *Hemidactylus saba*). In comparison, *Hemidactylus granosus* has a much wider distribution, spanning from northeastern Egypt to central Saudi Arabia. It was found from the sea-level up to almost 1600 m in the Asir Mountains, which stretch along the eastern Red Sea coast of the Arabian Peninsula. Its occurrence in eastern Egypt is also likely based on observations of Baha El Din (2005, 2006), who reported morphologically variable populations of *Hemidactylus turcicus* (sensu lato) in these regions attributable to *Hemidactylus granosus* (see Distribution and ecology). The distribution of *Hemidactylus granosus* in the coastal Sinai and Saudi Arabia near important marine junctions together with the genetic uniformity of this species indicates extensive gene flow between these populations. It may be the result of recent colonization event(s), their inadvertent human-mediated transportation or perpetual contact of populations in a continuous range. The continuous range of *Hemidactylus granosus* along the Hijaz and Asir Mountains in western Arabia confirms that these mountain ranges can serve as a corridor providing connection between the eastern Mediterranean and southern Arabia (Scott 1942; Gvoždík et al. 2010).

The highlands of southwestern Saudi Arabia and Yemen are known to host a high number of endemic taxa (Balletto et al. 1985; Arnold 1986; Gasperetti 1988; Harrison and Bates 1991; Gasperetti et al. 1993). The genus *Hemidactylus* also shows a high rate of speciation and endemicity in the area. Currently, there are eight species and one subspecies known from the Yemen highlands, which makes *Hemidactylus* one of the most specious reptile genera in the area (Fritz and Schütte 1987; Busais and Joger 2011b; Šmíd et al. 2013; Uetz 2013). As new genetic and morphological data are becoming available from Arabia even more new species are to be expected (Moravec et al. 2011; Šmíd et al. 2013), thus fulfilling the prognosis of Baha El Din (2005) and the models of Ficetola et al. (2013) which suggested that the Red Sea region is likely to contribute significantly to the diversity of *Hemidactylus*.

## Supplementary Material

XML Treatment for Hemidactylus granosus

XML Treatment for Hemidactylus ulii

## Appendix

### Specimens examined

*Hemidactylus flaviviridis* (8 individuals) - NMP6V 74858 (Oman, Jalan Bani Bu Hasan); NMP6V 74859/1–5 (Pakistan, Multan); NMP6V 74856 (Pakistan, Rakhni); NMP6V 74857 (Pakistan, Sukkur)

*Hemidactylus jumailiae* (18 individuals) - NMP6V 74818/1 (Yemen, near Al Bayda [At Dageeg]); NMP6V 74819 (Yemen, Sana’a); NHM-BS N41788, NHM-BS N41890 (paratype), NHM-BS N41891, NHM-BS N41893 (holotype), NHM-BS N41894 (paratype), NHM-BS N41897 (paratype) (Yemen, Ibb); NHM-BS N41898 (paratype, the same number as one of *Hemidactylus yerburii montanus* paratypes, Busais and Joger 2011b), NHM-BS N41899 (paratype) (Yemen, Thamar); BMNH1982.1143–44 (Yemen, Al Nabi Shuaib, 30 Km W. of Sana’a); BMNH1982.1145 (Yemen, Sana’a); BMNH1982.1146 (Yemen, Wadi Ahger, 45 Km. W. of Sana’a); BMNH1952.1.3.52 (Yemen, Sana’a); MSNG-YEM02, MSNG-YEM03 (Yemen, El Menghil); MCCI-R814 (Yemen, Hababah)

*Hemidactylus mindiae* (5 individuals) - NMP6V 71323/1–2 (Jordan, Jabal Ghazali); NMP6V 72739/1–3 (Jordan, Wadi Ramm Nughra Radet Salem)

*Hemidactylus robustus* (27 individuals) - SMF 8720 (lectotype), SMF 8721 (“Abyssinia” [Ethiopia and Eritrea]); SMF 8725–8726 – redetermined from *Hemidactylus granosus* (Egypt, Sinai); JS210, TMHC2012.07.092, TMHC2012.07.100 (Ethiopia, Jijiga), CAS130512 – redetermined from *Hemidactylus macropholis* as it is in the CAS catalogue (Kenya, vicinity of Mandera); NMP6V 74820 (Iran, Bandar Lengeh); NMP6V 74821/1–2 (Yemen, Wadi Zabid); NMP6V 74829 (Yemen, Bir Ali); JS144 (Kenya, Garissa); NMP6V 74867/1–3 (Oman, Muscat); NMP6V 74868 (Oman, Salalah); NMP6V 74869/1–7 (Oman, Mughsayl); NMP6V 74870/1–2 (Oman, Shisr); MCCI–R815 (Yemen, Zabid)

*Hemidactylus saba* (3 individuals) - NHM-BS N41912 (holotype, MorphoBank M305478–M305492), NHM-BS N41913 (paratype, MorphoBank M305493–M305504), NHM-BS N41914 (paratype, MorphoBank M305505–M305519) (Yemen, Marib)

*Hemidactylus sinaitus* (23 individuals) - BMNH82.8.16.27 (holotype, probably from Suakin, Sudan); BMNH97.10.28.83–85 (Sudan, Durrur, N of Suakin); BMNH97.10.28.87 (Sudan, Wadi Haifa); BMNH1974.3931 (Ethiopia, Mule River?, Danakil); BMNH1937.12.5.293–294 (Somalia, Borama district); BMNH95.5.23.7 (Yemen, Sheikh Osman, near Aden); BMNH1945.12.12.14 (Yemen, Bir Fadhl, Aden); NMP6V 74809/1–4 (Sudan, Wad Ben Naga); NMP6V 74810 (Sudan, 15 km SE Atbara); MZUF28645–646 (Yemen, Moka); MZUF10914, MSNM521 (Eritrea, Isola [island] Sheik-Said); MSNM523–524 (Eritrea, Ailet); CAS174021–022 (Sudan, Assalaya)

*Hemidactylus turcicus* (33 individuals) - NMP6V 34747 (Syria, Baniyas); NMP6V 34748/1–3 (Syria, Palmyra); NMP6V 34749 (Syria, Salkhad); NMP6V 70648/1–4 (Turkey, Kaş); NMP6V 70668 (Greece, Kastellorizo, St. Georgies); NMP6V 71056 (Egypt, Bahariya); NMP6V 71587/1–3 (Cyprus, Famagusta); NMP6V 71592/1–2 (Cyprus, Yali); NMP6V 72497 (Syria); NMP6V 74046/1–2 (Syria, Cyrrhus); NMP6V 74047/1–2 (Turkey, Antakya); NMP6V 74050 (Greece, Crete, Kavros); NMP6V 74131/1–3 (Syria, Palmyra); NMP6V 73626/1–3 (Turkey, Finike); NMP6V 70269 (Italy, Sardinia, Cagliari); NMP6V 72073 (Greece, Korfu, Nicos); NMP6V 74167 (Greece, Crete, Kavros); NMP6V 70667 (Greece, Kastellorizo); NMP6V 70163/5 (Egypt, Sharm el-Sheikh)

*Hemidactylus yerburii yerburii* (51 individuals) - NMP6V 74827/1–4 (Yemen, Jabel Habeshi); NMP6V 74825/1–2 (Yemen, Al Turbah); NMP6V 74826 (Yemen, N of Lahij, Wadi Tuban); NMP6V 74823/1–3 (Yemen, 14 km NW of Al Turbah); NMP6V 74824/1–2 (Yemen, 3 km S of Najd an Nashamah); NMP6V 74828/1–3 (Yemen, Al Hababi); NMP6V 74822/1–5 (Yemen, near Zinjubar); MSNG-YEM01 (Yemen, Ta’izz); MSNG-YEM05, MSNG-YEM06 (Yemen, Vahren); NHM-BS N41856–59, NHM-BS N41861–64, NHM-BS N41866, NHM-BS N41868–69, NHM-BS N41888 (Yemen, Tour Albaha); NHM-BS N41860 (Yemen, Lahij); NHM-BS N41871–72 (Yemen, Radfan); NHM-BS N41873 (Yemen, Shihr); NHM-BS N41875 (Yemen, Ariab); NHM-BS N41876–77, NHM-BS N41879–86 (Yemen, Lowder); NHM-BS N41887 (Yemen, Aden)

*Hemidactylus yerburii montanus* (57 individuals) - NMP6V 74802 (Yemen, Jabal Bura); NHM-BS N41751–52 (paratypes), NHM-BS N41758 (paratype), NHM-BS N41762–63, NHM-BS N41765–66, NHM-BS N41768–69, NHM-BS N41770 (paratype), NHM-BS N41772–74, NHM-BS N41779, NHM-BS N41783 (paratype), NHM-BS N41785 (paratype), NHM-BS N41791 (paratype), NHM-BS N41793 (paratype), NHM-BS N41797–800 (paratypes), NHM-BS N41802–06 (paratypes), NHM-BS N41807 (paratype), NHM-BS N41809 (paratype), NHM-BS N41811–15 (paratypes), NHM-BS N41818 (paratype), NHM-BS N41821 (paratype), NHM-BS N41823 (paratype), NHM-BS N41836 (holotype), NHM-BS N41839, NHM-BS N41840 (paratype), NHM-BS N41842 (paratype), NHM-BS N41843, NHM-BS N41844 (paratype), NHM-BS N41846, NHM-BS N41848, NHM-BS N41851–52, NHM-BS N41867 (paratype) (Yemen, Ibb); NHM-BS N41771 (paratype) (Yemen, Yareem); NHM-BS N41789–90 (Yemen, Thamar); NHM-BS N41833–34 (paratypes) (Yemen, Wadah); NHM-BS N41853–55 (paratypes) (Yemen, Sana’a).
